# TREM-1 Is Upregulated in Experimental Periodontitis, and Its Blockade Inhibits IL-17A and RANKL Expression and Suppresses Bone loss

**DOI:** 10.3390/jcm8101579

**Published:** 2019-10-01

**Authors:** Nagihan Bostanci, Toshiharu Abe, Georgios N. Belibasakis, George Hajishengallis

**Affiliations:** 1 Division of Oral Diseases, Department of Dental Medicine, Karolinska Institutet, 14104 Huddinge, Sweden; george.belibasakis@ki.se; 2 Center of Dental Medicine, University of Zürich, 8032 Zürich, Switzerland; 3 Department of Basic and Translational Sciences, School of Dental Medicine, University of Pennsylvania, Philadelphia, PA 19104, USA; toshiharu@ikeshita-abeshika.com (T.A.); geoh@upenn.edu (G.H.)

**Keywords:** TREM-1, periodontal disease, intervention, inflammation, LP17, IL-17, RANKL, OPG

## Abstract

Aim: Triggering receptor expressed on myeloid cells-1 (TREM-1) is a modifier of local and systemic inflammation. There is clinical evidence implicating TREM-1 in the pathogenesis of periodontitis. However, a cause-and-effect relationship has yet to be demonstrated, as is the underlying mechanism. The aim of this study was to elucidate the role of TREM-1 using the murine ligature-induced periodontitis model. Methods: A synthetic antagonistic LP17 peptide or sham control was microinjected locally into the palatal gingiva of the ligated molar teeth. Results: Mice treated with the LP17 inhibitor developed significantly less bone loss as compared to sham-treated mice, although there were no differences in total bacterial load on the ligatures. To elucidate the impact of LP17 on the host response, we analyzed the expression of a number of immune-modulating genes. The LP17 peptide altered the expression of 27/92 genes ≥ two-fold, but only interleukin (IL)-17A was significantly downregulated (4.9-fold). Importantly, LP17 also significantly downregulated the receptor activator of nuclear factor kappa-B-ligand (RANKL) to osteoprotegerin (OPG) ratio that drives osteoclastic bone resorption in periodontitis. Conclusion: Our findings show for the first time that TREM-1 regulates the IL-17A-RANKL/OPG axis and bone loss in experimental periodontitis, and its therapeutic blockade may pave the way to a novel treatment for human periodontitis.

## 1. Introduction

Periodontitis entails the destruction of the tooth-supporting (periodontal) tissues, as an outcome of their inflammatory response to the juxtaposed microbial biofilm forming on the tooth surface [1,2]. Although oral bacteria are essential for initiation of the disease, the resulting inflammation is what causes collateral damage to the tissues, which may eventually lead to tooth loss. The inflammatory mediators that lead to alveolar bone destruction form an intricate network [3,4], in which the receptor activator of NF-κB ligand (RANKL)/osteoprotegerin (OPG) system plays a crucial role as a terminal regulator of the resulting osteoclastogenesis and bone resorption [5,6]. Recently discovered host molecules, acting between the microbial challenge and the RANKL/OPG system, may lead to better understanding of the pathogenesis of periodontal disease and offer novel targets for therapeutic intervention. 

Triggering receptor expressed on myeloid cells 1 (TREM-1), a member of the immunoglobulin superfamily, has been defined as a modifier of local and systemic inflammation, especially in response to bacterial infections [7,8,9]. Bacterial infection can upregulate the membrane-bound and soluble forms of TREM-1, which in turn amplifies inflammation. This is a particularly crucial response associated with systemic sepsis [10,11]. Blockade of TREM-1 engagement by either soluble forms of TREM-1 or synthetic peptides thereof reduces hyper-inflammatory responses and morbidity [12]. In a TREM-1 knock-out mouse model of viral or parasitic infection, it was demonstrated that the lack of TREM-1 signaling mitigated the severity of inflammation and disease (as compared to the wild-type mice) without, however, affecting pathogen clearance [13]. The study by Weber et al. [13] suggested that TREM-1 regulates inflammation, and that its therapeutic targeting may be beneficial in infection-driven inflammatory diseases without compromising pathogen clearance. 

There is also correlative evidence to suggest that TREM-1 might modify periodontal inflammation. Specifically, the presence or expression of TREM-1 is increased in saliva, serum [14,15], gingival crevicular fluid [16,17,18], and gingival tissues [19] of patients with periodontitis as compared to individuals with periodontal health. TREM-1 levels also positively correlate with the levels of putative periodontal pathogens present in subgingival biofilms or lysed gingival tissue [16,19]. In this respect, multispecies biofilms [19] or *Porphyromonas gingivalis* alone induce TREM-1 gene expression in monocytes [20], whereas sub-antimicrobial doses of doxycycline can abolish this upregulatory effect [21].

The studies discussed above collectively suggest that TREM-1 expression is upregulated in periodontitis as a result of microbial stimulation. However, there are as-yet no interventional studies in preclinical models to conclusively demonstrate TREM-1 involvement in periodontitis. Hence, this in vivo study in a validated model of murine ligature-induced periodontitis [22] was designed to investigate the effect of local TREM-1 inhibition on the induction of experimental periodontitis, as well as on the expression of inflammation- and osteoclastogenesis-associated molecules in the gingival tissue. Our results described below implicate for the first time TREM-1 in the pathogenesis of periodontitis in a preclinical model and suggest a novel therapeutic approach for the treatment of this oral inflammatory disease.

## 2. Materials and Methods

### 2.1. Ligature-Induced Periodontitis Model in Mice 

The well-established ligature-induced periodontitis model in specific pathogen-free C57BL/6 mice was used as described earlier [22]. All animal procedures were performed according to protocols approved by the Institutional Animal Care and Use Committee of the University of Pennsylvania, and adequate measures were taken to minimize pain or discomfort. To induce experimental periodontitis, a 5-0 silk ligature was tied around the maxillary left second molar for up to 8 days (*n* = 4–5 mice/group). The unligated contralateral molar in each mouse was used as baseline control (unligated control). A synthetic peptide derived from the extracellular domain of TREM-1 (LP17; LQVTDSGLYRCVIYHPP, Pepscan, Lelystad, Netherlands) was used as described earlier [7]. The LP17 blocking peptide is considered as a competitive antagonist of membrane-bound TREM-1 for its natural ligand, therefore acting as a decoy receptor for TREM signaling [23]. For the intervention experiments performed in this study, 5 μg of LP17 peptide or PBS were injected into the palatal gingiva of the ligated second maxillary molar 1 days before placing the ligature and every day thereafter until the day before sacrifice (day 5).

The measurements on the alveolar bone height were done on defleshed maxillae under a Nikon SMZ800 microscope (Nikon Instruments, Melville, NY, USA), and images of the maxillae were captured using a Nikon Digital Sight DS-U3 camera controller. The distance between the cemento-enamel junction (CEJ) and alveolar bone crest (ABC) was measured at six predetermined points on the ligated molar and adjacent regions using NIS Elements software (Nikon Instruments, Melville, NY, USA) [22]. To calculate bone loss, the six-site total CEJ–ABC distance for the ligated side of each mouse was subtracted from the six-site total CEJ–ABC distance of the contralateral unligated side. The results are presented in millimeters, and negative values indicate bone loss relative to the unligated control.

### 2.2. Bacterial Counts on Silk Sutures

The ligated silk sutures obtained from LP17-treated or PBS-treated mice at day 5 were collected (*n* = 5 mice/group). These were suspended individually in sterile PBS, and adherent bacteria were disassociated from the sutures via high-speed vortexing for 2 min. Serial dilutions of the samples were plated onto blood agar plates (BD Difco Laboratories, Detroit, MI, USA), and the plates were incubated anaerobically at 37 °C for 7 days. Results are reported as the mean number of colony forming units (CFUs) per millimeter length of silk suture ± the standard error of the mean (SEM). Anaerobic CFUs were preferred over aerobic ones because of the stronger etiological association of anaerobic organisms with periodontitis.

### 2.3. Antimicrobial Effects of the Synthetic Peptides in Vitro

A 6-species oral biofilm model was used to investigate the potential antimicrobial effects of LP17. The biofilm consisted of *Actinomyces oris* OMZ 745, *Veillonella dispar* OMZ 493 (ATCC 17748T), *Fusobacterium nucleatum* OMZ 598 (KP-F2), *Streptococcus mutans* OMZ 918 (UA159), *Streptococcus oralis* OMZ 607 (SK 248), and *Candida albicans* OMZ 110. In brief, biofilms were grown according to the standard protocol in 24-well cell culture plates on sintered hydroxyapatite (HA) discs, which were pre-conditioned for 4 h with pooled human saliva, for pellicle formation. Throughout the following experimentation period, the biofilms were grown in the presence of LP17 or 0.9% NaCl (sham control). After 5 days of biofilm growth under anaerobic conditions, the HA discs were vortexed vigorously for 1 min in 1 mL of 0.9% NaCl and then sonicated (Branson Sonic Power Company, Danbury, CT, USA) for 5 s to harvest the adherent biofilms. Then, to determine the total CFUs, the bacterial suspensions were serially diluted in 0.9% NaCl and 50 μL aliquots were plated on agar plates supplemented with 5% whole human blood at 37 °C for 72 h.

### 2.4. Quantitative TaqMan Real-Time PCR and TaqMan Array Analysis

The TaqMan Array 96-well Mouse Immune Response kit (Applied Biosystems) was used to assess the expression of 92 predetermined genes mediating the immune response and four endogenous control genes including *GAPDH, HPRT, GUSB and 18S RNA*. For this analysis, gingival tissues were collected at day 5 (*n* = 3 mice/group). Total RNA was extracted from these tissues by Qiagen Fibrous Tissue Extraction kit. According to the manufacturer’s protocol, cDNA was mixed with 2× TaqMan Universal Master Mix and H_2_O to a total volume of 2160 μL. Subsequently, 20 μL of the mixture was placed into each well of the PCR array. The three steps of the cycling program were 95 °C for 10 min for 1 cycle, then 95 °C for 15 s, and 60 °C for 1 min for 40 cycles, using a Step One Plus Real-Time PCR System (Applied Biosystems). In addition, the transcription levels of TREM-1, interleukin (IL)-1β, RANKL, OPG, COX-2, and IL-6 were assessed by individual TaqMan Gene Expression Assays (Applied Biosytems). For TaqMan qPCR analysis, mouse ACTB (β-actin) was used as an endogenous control.

### 2.5. Statistical Analysis

All statistical analyses were performed using Prism v.6.0 software (GraphPad Software, La Jolla, CA, USA). One-way ANOVA was used to determine differences between three or more groups, whereas an unpaired, two-tailed Student *t* test was used to determine the statistical significance of differences between two groups. Differences were considered significant at *p* < 0.05.

## 3. Results

### 3.1. Local Tissue Kinetics of TREM-1 Expression in Ligature-Induced Periodontitis 

**Figure 1:**
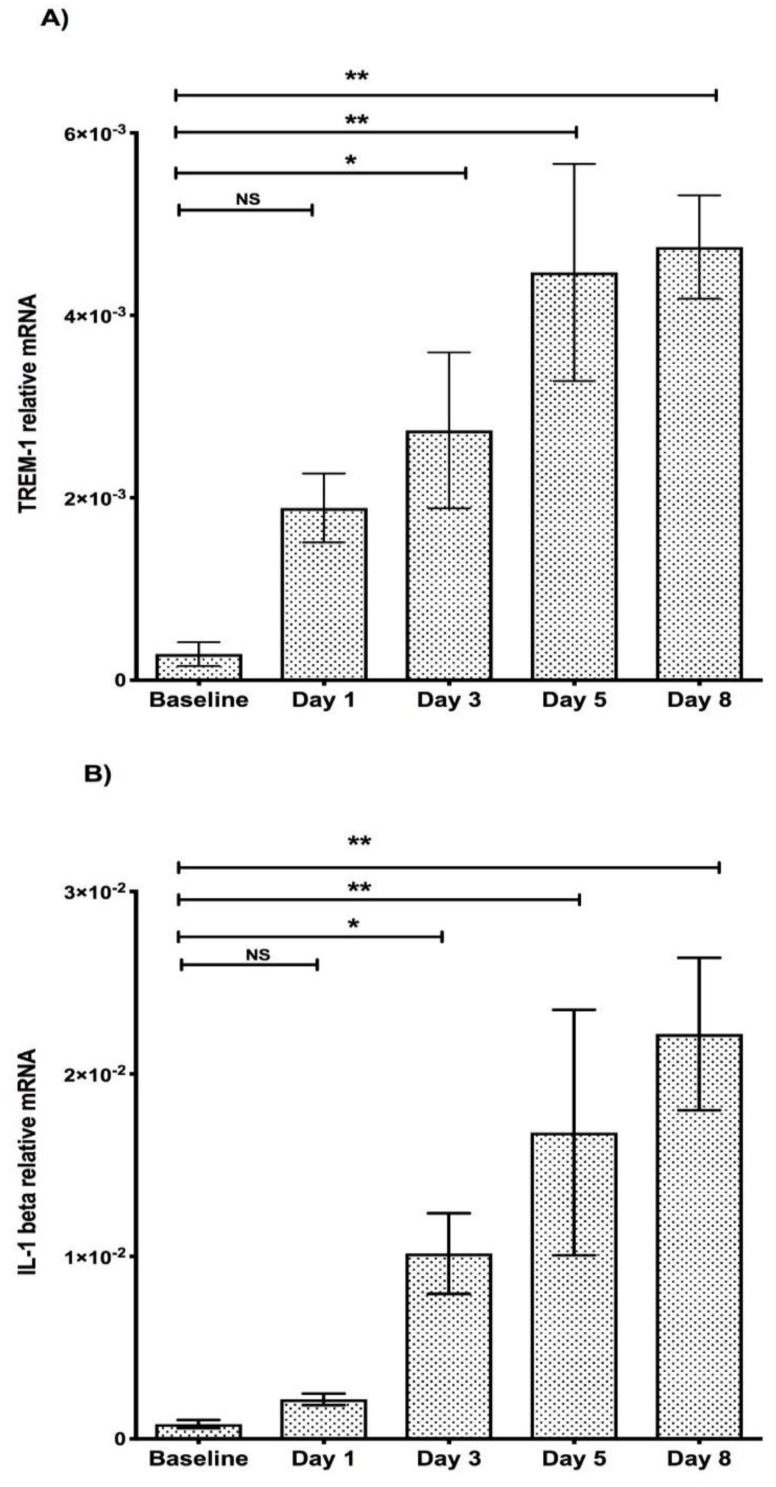
Kinetics of gingival tissue expression of TREM-1 and IL-1 beta in ligature-induced periodontitis. TREM-1 mRNA **(A)** and IL-1 beta mRNA **(B)** levels were examined in unligated control gingiva and ligature-induced periodontitis gingival tissues at 1 to 8 days. The gene expression levels were detected by TaqMan real-time qPCR and calibrated against the expression of housekeeping gene *β-actin*. Results are means ± SEM (*n* = 4 mice/group). * *p* < 0.05 and ** *p* < 0.01 between the indicated groups.

Using the ligature-induced murine periodontitis model, we first investigated the regulation of TREM-1 expression in the gingival tissue. TREM-1 gene expression was significantly upregulated at the ligated sites in a time-dependent manner, peaking at day 8 compared to the unligated control sites forming the baseline (Figure 1A). Compared to the unligated control sites, TREM-1 mRNA levels in the ligated sites were approximately 16-fold and 17-fold higher at day 5 and day 8, respectively (*p* < 0.01). Since TREM-1 activation is involved in the upregulation of a number of key proinflammatory cytokines [20], including IL-1β, which is crucial in periodontal pathogenesis, IL-1β gene expression levels were also assessed. A similar expression pattern was observed for IL-1β. In particular, compared to the baseline control, IL-1β gene expression at ligated sites was approximately 13-fold, 21-fold, and 27-fold higher at days 3, 5, and 8, respectively (*p* < 0.01) (Figure 1B).

### 3.2. Role of TREM-1 in Alveolar Bone Loss

**Figure 2:**
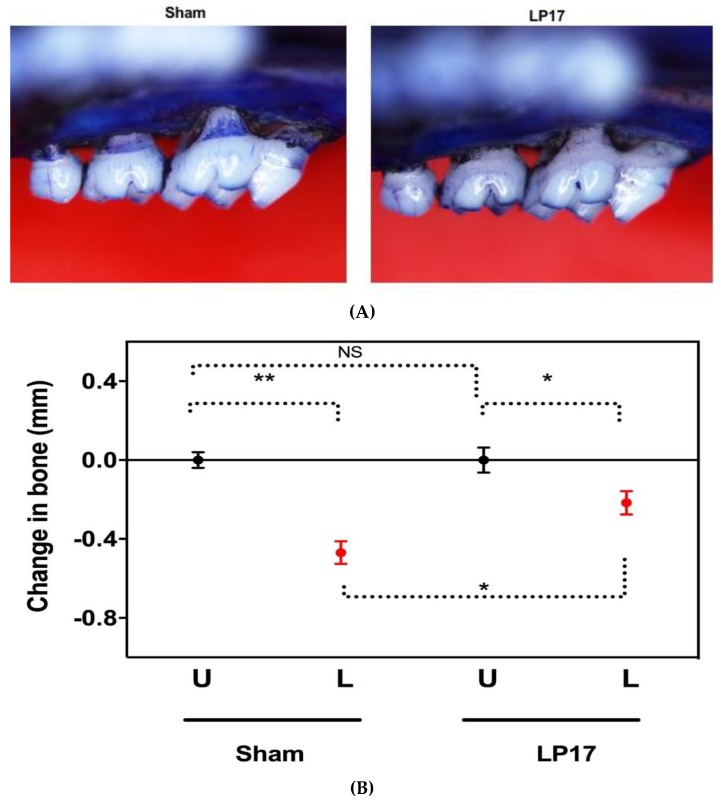
Inhibition of ligature-induced bone loss by LP17. Ligatures were placed on the left maxillary molars of C57BL/6 mice and then locally microinjected with 5 μg of TREM-1 blocking peptide (LP17) or with PBS sham 1 day before placing the ligature and every day thereafter until day 5. The distance between the cemento-enamel junction (CEJ) and alveolar bone crest (ABC) was measured at six predetermined points on the ligated side. Representative images of PBS sham- and LP17-treated maxillae exhibiting differential bone loss **(A).** To calculate bone loss, the six-site total CEJ–ABC distance for the ligated (L) side of each mouse was subtracted from the six-site total CEJ–ABC distance of the contralateral unligated (U) side. The results are presented in millimeters, and negative values indicate bone loss relative to the unligated control **(B)**. Data are means ± SEM (*n* = 4–5 mice/group). * *p* < 0.05 and ** *p* < 0.01 between the indicated groups.

The kinetics of TREM-1 gene expression followed a pattern similar to those of ligature-induced bone loss seen in our previous publication [22]. To determine whether there was a cause-and-effect relationship between gingival TREM-1 expression and alveolar bone loss, we subjected groups of mice to ligature-induced periodontitis with local administration of the LP17 or with PBS sham control. Five days after placement of the ligatures, mice treated with LP17 developed significantly less alveolar bone loss as compared to the sham-treated mice (*p* < 0.05) (Figure 2A,B), indicating that TREM-1 signaling contributes to induction of alveolar bone loss. 

### 3.3. Investigation of Potential LP17 Antimicrobial Activity

**Figure 3:**
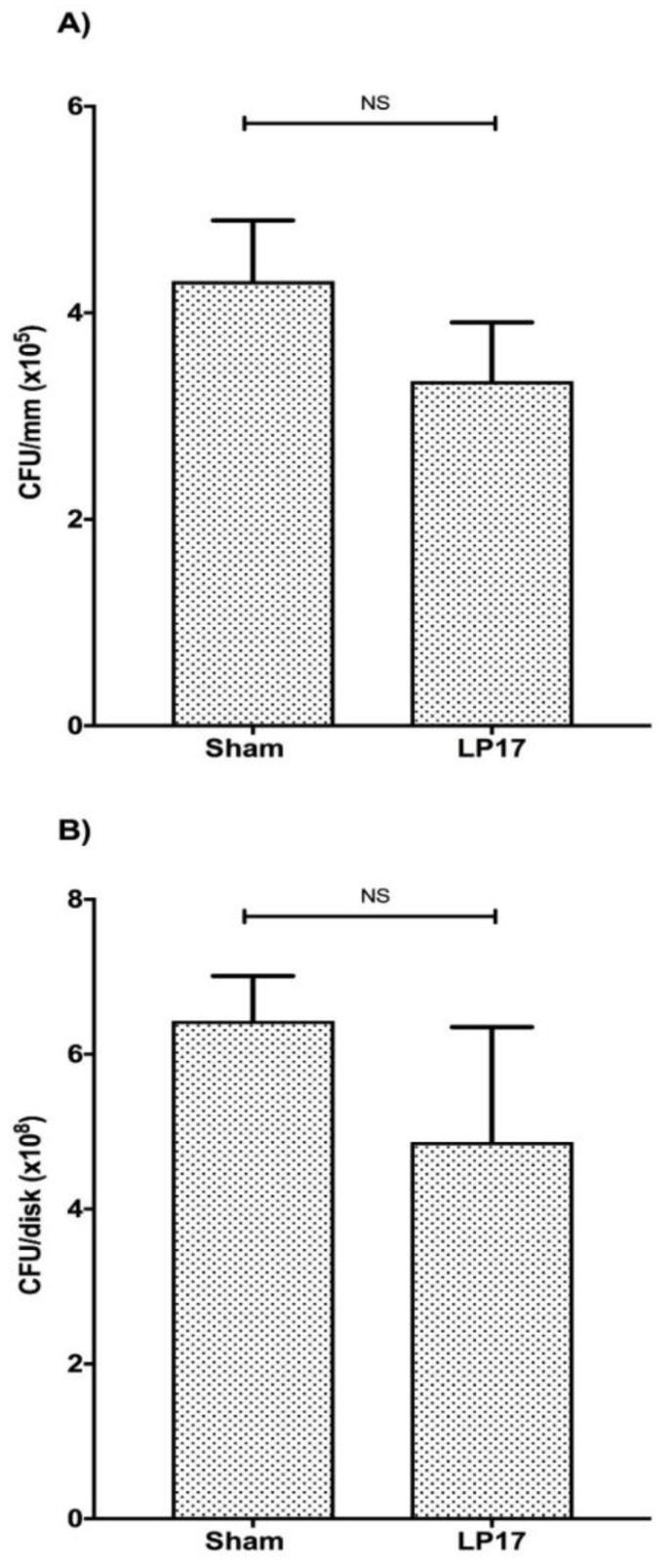
LP17 does not affect the microbial load in vivo. (**A)** Detached material from the recovered ligatures at day 5 from mice used in Figure 2 were cultivated anaerobically for 7 days on blood agar plates, followed by total colony forming unit (CFU) determination. To normalize the data, the counted CFUs were divided by the length of corresponding suture. Data are means ± SEM (*n* = 5 mice/group). NS: Not significant, *p* > 0.05. (**B**) LP17 does not affect the microbial load in vitro. The in vitro biofilms were grown in the presence of LP17 or 0.9% NaCl (sham). After 5 days of biofilm growth under anaerobic conditions, biofilm bacteria were harvested from the discs and cultivated anaerobically for 3 days on blood agar plates, followed by total CFU determination. The CFUs are given per hydroxyapatite (HA) disc. Data are means ± SEM (*n* = 3 disc /group). NS: Not significant, *p* > 0.05.

To determine whether the protective effects of LP17 in ligature-induced periodontitis could, in part, be attributed to potential antimicrobial effects, we determined the microbial load of the treated mice from the above-described in vivo experiment (Figure 2). To this end, bacteria were extracted from the recovered ligatures (day 5) and cultivated anaerobically for 7 days on blood agar plates. To normalize the data, the counted CFUs were divided by the length of corresponding suture, and the results revealed that sutures from the LP17-treated mice yielded similar CFUs, as compared to the PBS sham-treated group (*p* > 0.05) (Figure 3A). Furthermore, the potential antimicrobial impact of LP17 was tested in vitro on a 6-species biofilm model for 5 days. A 1.3-fold reduction in total CFUs was observed, compared to the saline sham-treated group (*p* > 0.05). These results suggested that the LP17 peptide preferentially acted by regulating the host response rather than bacterial growth. Hence, we next investigated the host-modulating activity of LP17.

### 3.4. Modulation of Immunoregulatory Genes by TREM-1 

**Figure 4:**
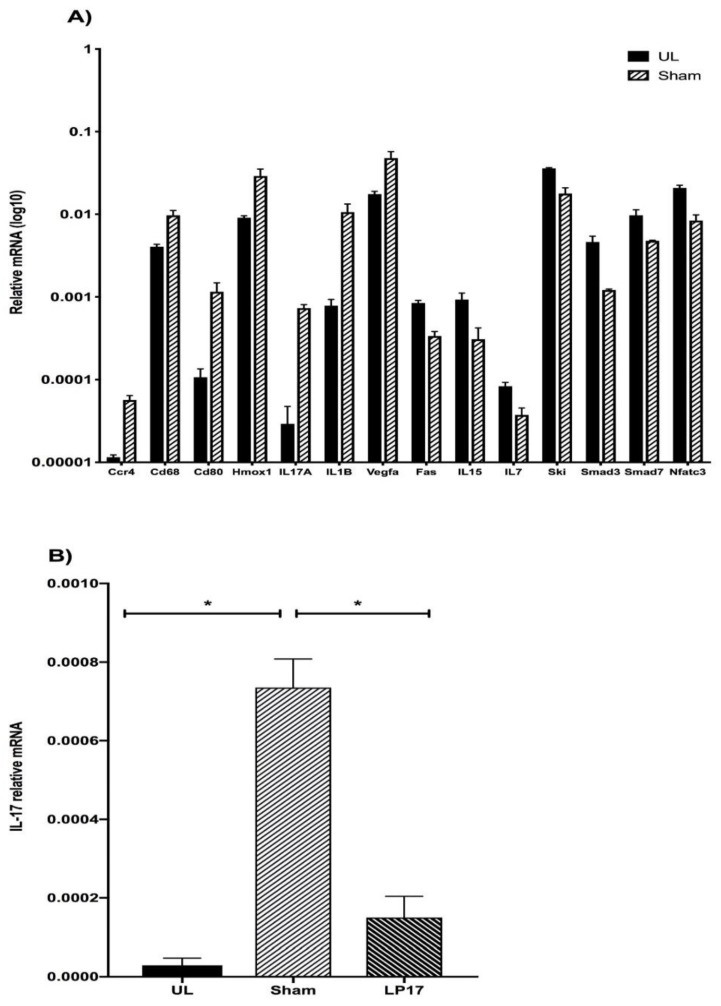
Modulation of immunoregulatory genes by TREM-1. Dissected gingiva from unligated control sites (UL) and ligated shame treated sites (Sham) for 5 days. The mRNA expression of 92 key genes mediating the immune response and four endogenous control genes including *GAPDH, HPRT, GUSB and 18S RNA mRNA* were assessed by qPCR. The gene expression levels were calibrated against the expression of housekeeping genes (detailed list provided in Table S2). The significantly regulated genes are presented (fold-change ≥ 2 and * *p* < 0.05). **(A).** Dissected gingiva from unligated control sites (UL), or ligated sites from PBS sham-treated sites (Sham) or sites treated with 5 μg synthetic TREM-1 inhibitor (LP17) for 5 days. The mRNA expression of IL-17 is presented (fold-change ≥ 2 and * *p* < 0.05). **(B)**. Data are means ± SEM (*n* = 3 mice/group).

To understand how TREM-1 signaling regulates the host periodontal response, the defleshed gingival tissues were analyzed for the expression of a number of immunoregulatory genes at day 5, by using a mouse immune response qPCR Array profiling for 92 individual genes (Table S1). Differential expression analysis was done by the following pair-wise comparisons: (a) unligated sites versus ligated sites and (b) PBS sham-treated ligated sites versus LP17-treated ligated sites. Although a basal expression level was detected for all studied genes at unligated control sites, a total of 38 genes were differentially transcribed by more than two-fold during the experimental infection period (Table S1). Among those, 27 genes were induced in the ligature-induced gingival sites, 7 of which reached statistical significance (*p* < 0.05). Another 11 genes were repressed more than two-fold, 7 of which also reached statistical significance (*p* < 0.05). The significantly upregulated genes were *IL-17A, IL-1β, CD80, CCR4, HMOX1, VEGFA,* and *CD68*, whereas the significantly downregulated ones were *SKI, SMAD 7, IL-7, NFATC 3, FAS, IL-15,* and *SMAD 3* (Figure 4A).

Treatment with the LP17 peptide altered the expression of 27 genes by more than two-fold (23 downregulated, 4 upregulated) (Supplement Table S2). Although the expression of proinflammatory cytokines associated with periodontal disease pathogenesis (such as, IL-1β, IL-6, IL-17A, and TNF) was inhibited, statistical significance was reached only for IL-17A, which was downregulated by 4.9-fold (Figure 4B). Taken together, these data indicate that ligature-induced periodontitis is associated with upregulation of a number of proinflammatory genes that seem to be inhibited by LP17, which predominantly targets IL-17A expression, a signature cytokine of Th17 cells that were shown to drive inflammatory bone loss in mice and humans [24]. 

### 3.5. Regulation of the RANKL/OPG Axis by TREM-1

**Figure 5:**
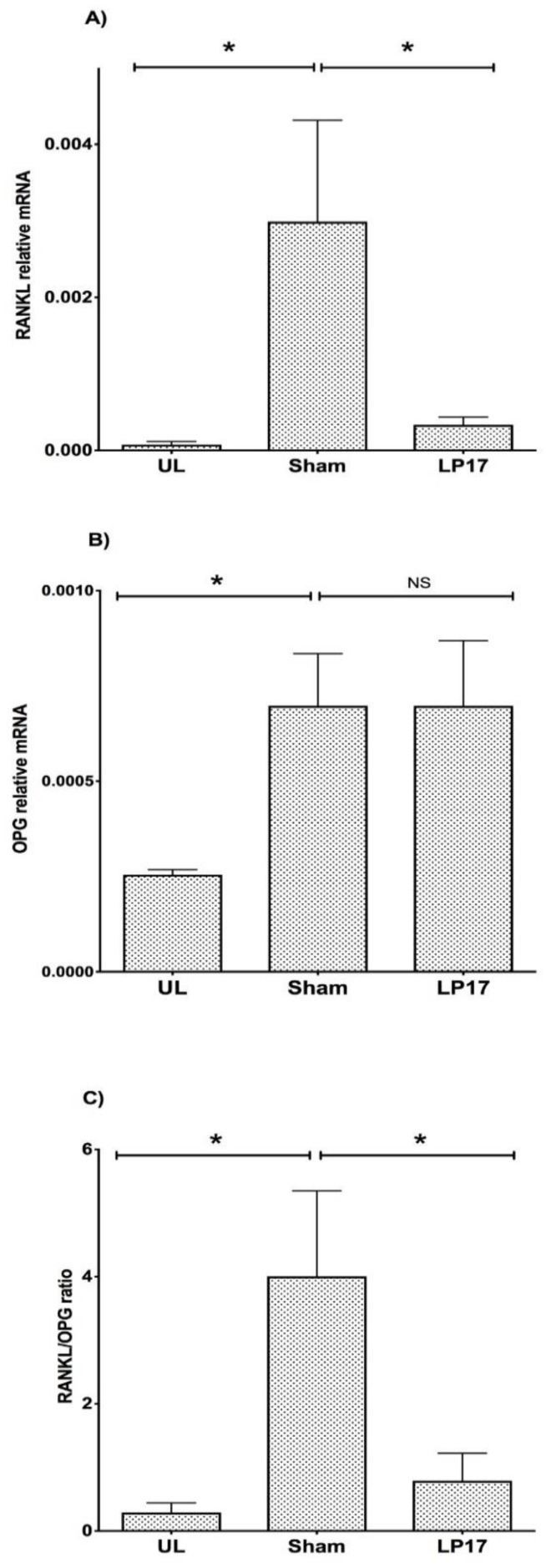
Inhibition of receptor activator of nuclear factor kappa-B-ligand (RANKL)/osteoprotegerin (OPG) ratio by LP17. Gingival tissue samples were dissected at day 5 from mice used in Figure 2 and were processed for gene expression of RANKL **(A)** and OPG **(B)** by qPCR. The relative RANKL/OPG ratio was also calculated **(C)**. The expression of the indicated molecules was determined in unligated (UL) control gingiva and in ligated gingival tissues treated with 5 μg synthetic TREM-1 inhibitor (LP17) or PBS sham. The gene expression levels were detected by TaqMan real-time qPCR and calibrated against the expression of the housekeeping gene *β-actin*. Results are means ± SEM (*n* = 4 mice/group). * *p* < 0.05 and ** *p* < 0.01 between the indicated groups. NS: Not significant, *p* > 0.05.

The upregulation of IL-17A expression, a cytokine associated with chronic inflammatory tissue destruction and alveolar bone loss [25,26], prompted us to investigate further the involvement of TREM-1 in the molecular regulatory mechanisms of bone resorption, particularly the RANKL/OPG system. RANKL was significantly induced at the sham-treated ligated sites (39-fold), whereas administration of LP17 inhibited this upregulatory effect by 8.9-fold (Figure 5A). The expression of OPG also increased at the sham-treated ligated sites (2.7-fold) but was not significantly affected by administration of LP17 (Figure 5B). As a result, the relative RANKL/OPG ratio, a molecular determinant of bone resorption, was significantly reduced in response to LP17 treatment by five-fold as compared to PBS sham treatment (Figure 5C). 

**Figure 6:**
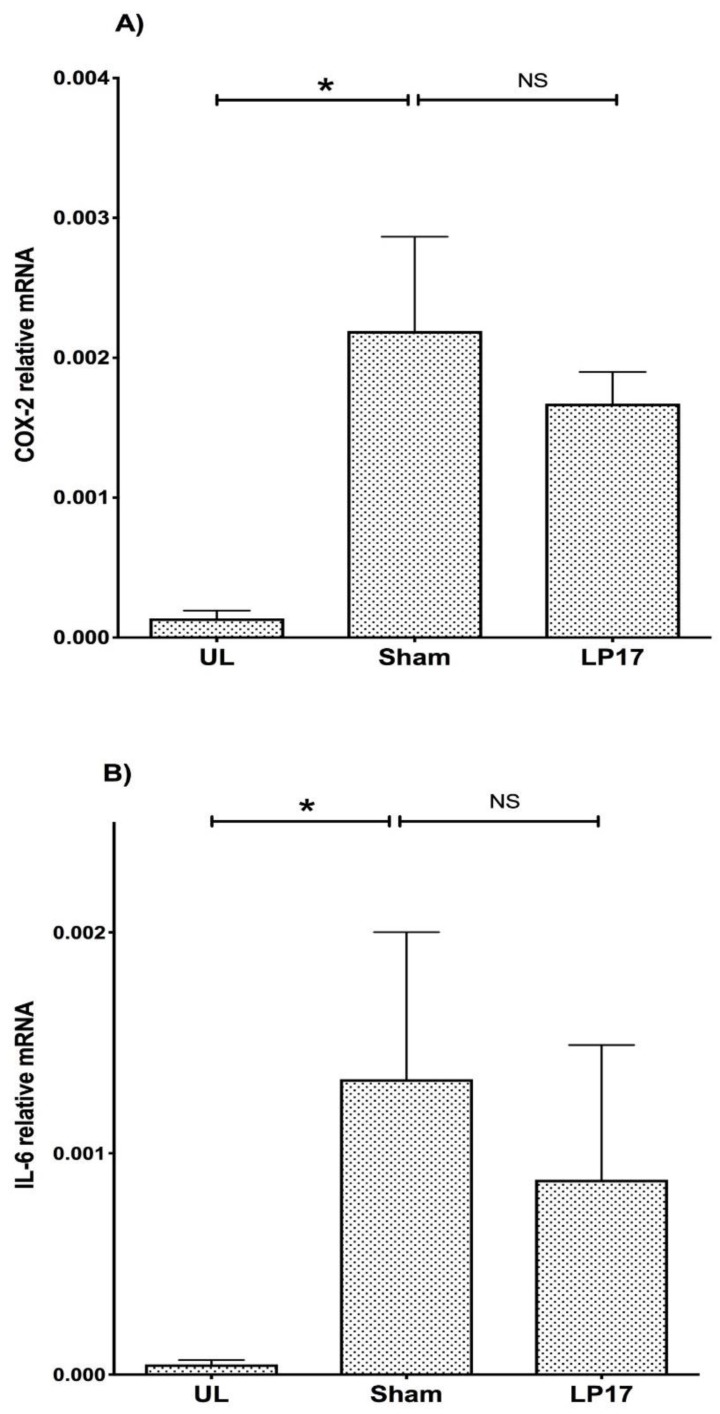
LP17 does not affect COX-2 and IL-6 levels. Gingival tissue samples were dissected at day 5 from mice used in Figure 2 and were processed for gene expression of COX-2 **(A)** and IL-6 **(B)** by qPCR. The expression of the indicated molecules was determined in unligated (UL) control gingiva and in ligated gingival tissues treated with 5 μg synthetic TREM-1 inhibitor (LP17) or PBS sham. The gene expression levels were detected by TaqMan real-time qPCR and calibrated against the expression of the housekeeping gene *β-actin*. Results are means ± SEM (*n* = 4 mice/group). NS: Not significant, *p* > 0.05.

As IL-6 and COX-2 that are produced in high levels during inflammation are considered as key regulators of RANKL expression [4,27,28], we assessed their regulation by TREM-1. Interestingly, the expressions of COX-2 and IL-6 were not significantly affected by LP17 treatment, indicating that the regulation of RANKL in this model may not be dependent on COX-2 (Figure 6A) or IL-6 (Figure 6B).

## 4. Discussion

Our present study shows for the first time that TREM-1 regulates alveolar bone loss in experimental periodontitis and paves the way for a novel approach to treat human periodontitis. In line with earlier observations in humans demonstrating upregulated TREM-1 gingival expression in periodontitis patients, as compared to healthy controls [19], our study showed progressively increased induction of TREM-1 gingival expression during the course of experimental periodontitis in response to biofilm accumulation. TREM-1 propagates proinflammatory cytokine expression, representatively demonstrated by IL-1β in the present study. These findings are in accordance with our previous studies showing a positive correlation between subgingival biofilms and TREM-1 levels in gingival tissue or gingival crevicular fluid of individuals with periodontitis [16,19]. Although the cellular distribution of TREM-1 in gingival tissue was not investigated in the present experimental setting, monocytes/resident macrophages and polymorphonuclear neutrophilic leukocytes (PMNs) are known to be a major source of TREM-1 in inflammation [9,29,30,31]. In this respect, our earlier work presented that multispecies oral biofilms [19] or the keystone pathogen *Porphyromonas gingivalis* is able to induce TREM-1 gene expression in monocytes or in PMNs in the tissue culture systems [20,32]. 

The major novel finding of this in vivo study is that local (gingival) injection of a TREM-1 blocking peptide, namely LP17, substantially reduced the RANKL/OPG osteoclastogenic ratio and alveolar bone loss, thus providing preclinical support for a new therapeutic target for periodontitis. Clinical studies have demonstrated that the RANKL/OPG ratio as well as IL-17 gingival tissue expression are upregulated in human periodontitis [33,34,35,36]. Intriguingly, LP17 selectively downregulated IL-17 expression among all studied immune response markers. Given that IL-17 regulates the expression of RANKL [37], it is possible that the capacity of LP17 to downregulate the RANKL/OPG ratio may be mediated through its ability to inhibit IL-17. Similarly, inhibition of IL-17 by its antagonist Del-1 has been shown to efficiently block osteoclastogenesis and subsequent periodontitis [38,39]. Thus, our study lends further support to the concept that IL-17 is a key driver of periodontal bone loss, although this is the first time that TREM-1 signaling is linked to IL-17 in the context of periodontitis.

Our present data are also consistent with a recent in vitro study, demonstrating that the LR12 TREM-1 inhibitor LR12 could prevent monocytic activation by *P. gingivalis* LPS [40], as well as our earlier in vitro findings demonstrating that LP17 can reduce cytokine release by monocytes in response to *P. gingivalis* whole bacteria [20,21]. Moreover, an earlier study in a psoriasis model showed that TREM-1 blockade in vitro and ex vivo significantly reduced the number of Th17 cells and decreased the secretion of IL-17, suggesting that TREM-1 positively regulates Th17 responses [41]. The COX-2 pathway and IL-6 are also important regulators of the RANKL/OPG ratio [27]. However, it is unlikely that TREM-1 regulates the RANKL/OPG ratio via COX-2 or IL-6 since LP17 failed to affect the expression of either gene. Thus, it is concluded that the alveolar bone resorptive effects of TREM-1 are, at least in part, mediated through activation of the IL-17-RANKL axis.

Although the impact of TREM-1 signaling on microbial control has been controversial in several bacterial challenge models [13], in the experimental periodontitis model, LP17 did not show a significant effect on oral bacterial load. This finding suggests that TREM-1 inhibition protects against periodontitis predominantly through host-modulation effects and is in line with earlier work indicating that LP17 did not alter the in vitro levels of *P. gingivalis* [20,32,42]. 

Moreover, TREM-1-deficient mice used in colitis and other experimental models of infection-driven inflammatory diseases exhibited no alterations in microbial clearance efficiency [13]. On the other hand, studies on lung infection models (e.g., *Pseudomonas aeruginosa*-induced pneumonia) indicated that administration of LP17 peptide reduced the bacterial load at an early stage of infection while increasing it at later stages; these effects, however, were attributed to indirect antimicrobial effects of TREM-1 related to early enhancement of neutrophil influx and consequent increase in phagocytic activity [43]. These observations are in line with the main function of TREM-1 as an inflammation fine-tuner [44], rather than a direct eliminator of infection, as is the case, for instance, for TNF-alpha. Yet, the use of anti-TNF antibody may be complicated because of the increased risk for reactivation of latent infection [45].

## 5. Conclusions

The present study conclusively demonstrated the involvement of TREM-1 in alveolar bone resorption during the course of experimental periodontitis in mice. Mechanistically, TREM-1 reduced the RANKL/OPG osteoclastogenic ratio, presumably via the inhibition of IL-17. Importantly, our findings also reveal a previously unidentified TREM-1-driven axis for inflammatory bone loss that could be targeted via small-molecule antagonists for therapeutic intervention in human periodontitis. 

## Supplementary Materials

The following are available online at https://www.mdpi.com/2077-0383/8/10/1579/s1, Table S1: Immune gene expression profile in ligated gingiva vs healthy gingiva, Table S2: immune regulatory genes in mouse gingiva by LP17.
