# Supplementary material for: TREM-1 Is Upregulated in Experimental Periodontitis, and Its Blockade Inhibits IL-17A and RANKL Expression and Suppresses Bone loss
Source: J Clin Med. 2019 Oct 1;8(10):1579. doi: 10.3390/jcm8101579 (PMC6832657; doi:10.3390/jcm8101579)
Supplement: Supplementary file 1 [file jcm-08-01579-s001.pdf]

Supplement File

Table 1. Immune gene expression profile in ligated gingiva vs healthy gingiva.

Gene name	Fold-change	Down or Up Regulation
**IL-17a**	25.21*	**Up**
**Ccr7**	21.25	
**Ccl3**	17.08	
**Ptgs2**	16.81	
**IL-1b**	13.56*	**Up**
**Csf3**	11.67	
**Cd80**	10.84*	**Up**
**IL-6**	9.87	
**IL-2ra**	8.75	
**IL-10**	6.14	
**Ccr4**	4.91*	**Up**
**IL-13**	4.81	
**IL-1a**	4.64	
**Gzmb**	4.56	
**Tnf**	4.35	
**Ccl5**	4.25	
**Ccl2**	4.23	
**Lif**	3.82	
**Csf2**	3.64	
**Hmox1**	3.20*	**Up**
**Ctla4**	2.92	
**Icos**	2.88	
**Vegfa**	2.72*	**Up**
**Cd3e**	2.55	
**Cxcl11**	2.54	
**Cd68**	2.40*	**Up**
**Cxcl10**	2.35	
**Stat4**	1.92	
**Cd86**	1.90	
**Edn1**	1.89	
**IL-5**	1.82	
**Ptprc**	1.80	
**Nfkb2**	1.76	
**Prf1**	1.71	
**Vcam1**	1.69	
**Ccr2**	1.66*	
**Agtr2**	1.64	
**Socs1**	1.51*	
**Selp**	1.51	
**Stat3**	1.50	
**IL-12b**	1.46	
**Tnfrsf18**	1.44	
**Cd38**	1.33	
**H2-Eb1**	1.32	
**Lrp2**	1.25	
**Cd40**	1.20	

**IL-2**	1.17	
**Bax**	1.12	
**Socs2**	1.07	
**Cd19**	0.99	
**Cd40lg**	0.99	
**Cyp1a2**	0.99	
**Cyp7a1**	0.99	
**Fasl**	0.99	
**H2-Ea**	0.99	
**Ifng**	0.99	
**IL-12a**	0.99	
**IL-3**	0.99	
**IL-4**	0.99	
**IL-9**	0.99	
**Lta**	0.99	
**Nfkb1**	0.95	
**Icam1**	0.95	
**Stat6**	0.95	
**Csf1**	0.94	
**Cd8a**	0.93	
**Bcl2**	0.91	
**Nos2**	0.90	
**Stat1**	0.88	
**Nfatc4**	0.77	
**Tbx21**	0.75	
**Cd4**	0.75	
**Cd34**	0.73	
**Ccl19**	0.70	
**Bcl2l1**	0.67*	
**Tgfb1**	0.65*	
**Cd28**	0.63	
**Ikbkb**	0.58	
**Ly96**	0.57	
**Ace**	0.53	
**Sele**	0.50	
**Ski**	0.49*	**Down**
**Smad7**	0.49*	**Down**
**IL-7**	0.44*	**Down**
**Cxcr3**	0.42	
**Fn1**	0.40	
**Nfatc3**	0.40*	**Down**
**Fas**	0.39*	**Down**
**IL-18**	0.40	
**IL-15**	0.32*	**Down**
**Smad3**	0.26*	**Down**
**C3**	0.22	

Dissected gingiva from unligated control sites and ligated sites at day 5 were obtained and the mRNA expression of 92 key genes mediating the immune response and four endogenous control genes including GAPDH, HPRT, GUSB and 18S RNA mRNA were assessed. The gene expression levels were calibrated against the expression of housekeeping genes. Data are means ± SEM (*n* = 3 mice/group). Fold changes were calculated from the following comparisons: the ligated sites Vs unligated control sites. The data is sorted by magnitude of the fold-change. A gene was considered regulated if the fold-change difference was ≥2 and *p<0.05.

Supplement Table S2. immune regulatory genes in mouse gingiva by LP17.

Gene name	Fold-change	Down or Up egulation
**IL-17a**	4.90*	Down
**Cd68**	1.84*	Down
**H2-Eb1**	1.82*	Down
**Lif**	12.63	
**Csf2**	8.68	
**Cxcl11**	7.46	
**Ctla4**	5.41	
**IL-13**	4.86	
**Ccl3**	4.58	
**Ccl5**	3.79	
**Stat4**	3.51	
**Ccl19**	3.04	
**C3**	2.89	
**Cd80**	2.81	
**Ccl2**	2.81	
**Vcam1**	2.77	
**Csf3**	2.55	
**Cd38**	2.50	
**IL-6**	2.44	
**Fn1**	2.40	
**IL-2ra**	2.25	
**IL-10**	2.24	
**Tnf**	2.19	
**Edn1**	2.14	
**Ccr4**	2.13	
**IL-1b**	1.87	
**Tbx21**	1.85	
**IL-5**	1.84	
**Cd40**	1.79	
**Ccr7**	1.72	
**Ptprc**	1.54	
**Csf1**	1.51	
**Vegfa**	1.50	
**Ptgs2**	1.50	
**IL-12b**	1.48	
**IL-1a**	1.44	
**Gzmb**	1.41	
**Nfatc4**	1.41	
**Cd34**	1.34	
**Icos**	1.30	
**Selp**	1.30	
**Icam1**	1.28	
**Bcl2**	1.27	
**Nos2**	1.25	
**Hmox1**	1.24	
**Socs1**	1.19	
**Cd4**	1.18	
**IL-2**	1.18	
**Tgfb1**	1.14	
**Prf1**	1.12	

**Socs2**	1.12
**Cxcl10**	1.11
**Cd86**	1.09
**Ccr2**	1.09
**Ace**	1.05
**Smad7**	1.02
**Cxcr3**	1.01
**Cd19**	1.00
**Cyp1a2**	1.00
**Cyp7a1**	1.00
**H2-Ea**	1.00
**Ifng**	1.00
**IL-12a**	1.00
**IL-3**	1.00
**IL-4**	1.00
**IL-9**	1.00
**Lta**	1.00
**Bcl2l1**	0.97
**Stat3**	0.97
**Fas**	0.97
**Nfkb1**	0.96
**Bax**	0.95
**Nfkb2**	0.94
**Stat6**	0.91
**Agtr2**	0.91
**Smad3**	0.90
**IL-7**	0.89
**Tnfrsf18**	0.87
**Ikbkb**	0.85
**Ski**	0.84
**Lrp2**	0.79
**Sele**	0.77
**Nfatc3**	0.74
**Cd40lg**	0.74
**Stat1**	0.71
**Cd28**	0.69
**Cd3e**	0.64
**Ly96**	0.60
**Cd8a**	0.43
**IL-15**	0.39
**IL-18**	0.35
**Fasl**	0.28

Dissected gingiva was obtained from PBS sham treated sites or sites treated with an antagonistic synthetic peptide (LP17). The mRNA expression of 92 key genes mediating the immune response and four endogenous control genes including GAPDH. HPRT. GUSB and 18S RNA mRNA were assessed by qPCR. The gene expression levels were calibrated against the expression of housekeeping genes. Data are means ± SEM (n = 3 mice/group) and *$p < 0.05$. Fold changes were calculated from the following comparisons: LP17 Vs PBS sham. The data is sorted by the fold change differences in descending order. A gene was considered regulated if the fold-change difference was ≥2 and *p<0.05.
